# The role of general practice in routes to diagnosis of lung cancer in Denmark: a population-based study of general practice involvement, diagnostic activity and diagnostic intervals

**DOI:** 10.1186/s12913-014-0656-4

**Published:** 2015-01-22

**Authors:** Louise Mahncke Guldbrandt, Morten Fenger-Grøn, Torben Riis Rasmussen, Henry Jensen, Peter Vedsted

**Affiliations:** Research Centre for Cancer Diagnosis in Primary Care, Research Unit for General Practice, Aarhus University, Bartholins Alle 2, 8000 Aarhus, Denmark; Section for General Medical Practice, Department of Public Health, Aarhus University, Aarhus, Denmark; Department of Respiratory Diseases and Allergy, Aarhus University Hospital, Aarhus, Denmark

**Keywords:** Lung cancer, Cancer pathways, General practice, X-ray, Diagnostic intervals, Denmark

## Abstract

**Background:**

Lung cancer stage at diagnosis predicts possible curative treatment. In Denmark and the UK, lung cancer patients have lower survival rates than citizens in most other European countries, which may partly be explained by a comparatively longer diagnostic interval in these two countries. In Denmark, a pathway was introduced in 2008 allowing general practitioners (GPs) to refer patients suspected of having lung cancer directly to fast-track diagnostics. However, symptom presentation of lung cancer in general practice is known to be diverse and complex, and systematic knowledge of the routes to diagnosis is needed to enable earlier lung cancer diagnosis in Denmark. This study aims to describe the routes to diagnosis, the diagnostic activity preceding diagnosis and the diagnostic intervals for lung cancer in the Danish setting.

**Methods:**

We conducted a national registry-based cohort study on 971 consecutive incident lung cancer patients in 2010 using data from national registries and GP questionnaires.

**Results:**

GPs were involved in 68.3% of cancer patients’ diagnostic pathways, and 27.4% of lung cancer patients were referred from the GP to fast-track diagnostic work-up. A minimum of one X-ray was performed in 85.6% of all cases before diagnosis. Patients referred through a fast-track route more often had diagnostic X-rays (66.0%) than patients who did not go through fast-track (49.4%). Overall, 33.6% of all patients had two or more X-rays performed during the 90 days before diagnosis. Patients whose symptoms were interpreted as non-alarm symptoms or who were not referred to fast-track were more likely to experience a long diagnostic interval than patients whose symptoms were interpreted as alarm symptoms or who were referred to fast-track.

**Conclusions:**

Lung cancer patients followed several diagnostic pathways. The existing fast-track pathway must be supplemented to ensure earlier detection of lung cancer. The high incidence of multiple X-rays warrants a continued effort to develop more accurate lung cancer tests for use in primary care.

## Background

Lung cancer is a significant health problem worldwide. It is currently the most common cause of cancer death in the developed world [1], and earlier diagnosis may promote curative treatment. Danish lung cancer patients have very low survival rates compared with patients from comparable countries [2]. In 2005–2007, the one-year relative survival for all lung cancers was 35% in Denmark compared with 44% in Sweden [2]. This figure could be explained by later diagnosis of lung cancer in Denmark as increased waiting time and diagnostic delay are generally believed to allow tumour stage progression. Danish patients’ survival deficit may be related to the degree of cancer awareness and the extent of diagnostic activity at the level of primary care. This calls for further investigation of the diagnostic pathway in Danish lung cancer patients.

A national strategy to improve cancer outcome in Denmark was introduced in 2008, including, among others, a fast-track diagnostic pathway for suspected cancer cases; this implied that patients with specific symptoms (e.g. sustained coughing) were to be seen at the hospital within three days after referral [3,4].

Most lung cancer patients present with clinical symptoms before they are diagnosed with cancer [5], and evidence suggests that symptoms are often experienced long before the diagnosis is made [6,7]. Yet, most lung symptoms do not represent underlying cancer, and the general practitioner (GP) must therefore interpret the symptoms and weigh the small risk of underlying cancer against the likelihood of the symptoms reflecting a benign or self-limiting illness [8] and on this basis decide on the appropriate level of diagnostic work-up.

Apart from clinical skills, the GP’s principal diagnostic tool for lung symptoms is a chest X-ray. This tool is, however, not very accurate, and a false negative X-ray may even increase the diagnostic interval as about 20% of all lung cancer patients have false negative chest X-rays before diagnosis [9-11].

A British study found that only 23% of 409 included lung cancer patients followed the route from symptom presentation in general practice to fast-track referral, while the rest obtained their diagnosis through other routes [12]. In order to facilitate earlier lung cancer diagnosis in general practice, we need to know more about the GPs’ symptom interpretation, their diagnostic activity and lung cancer patients’ diagnostic pathways.

The aim of this paper was to describe Danish patients’ different routes to diagnosis of lung cancer in general and the pre-diagnostic activities undertaken by GPs in particular. In addition, we aimed to explore the diagnostic intervals for specific patient groups.

## Methods

We conducted a national cohort study on first-time lung cancer patients, using data from national registers and questionnaires filled in by GPs. The civil registration number (CRN), a unique 10-digit personal identification number assigned to every Danish citizen, was used to link registers [13].

### Setting

The study took place in Denmark in 2010. Danish GPs act as gatekeepers to the rest of the health-care system, except for emergencies; and 99% of all Danish citizens are registered with a general practice which they consult for medical advice.

### Study population

Patients were identified in the Danish National Patient Registry (NPR) [14]. The NPR is a national population-based database containing admission and discharge dates for all Danish citizens treated at Danish hospitals; and all recorded diagnoses are classified according to the International Classification of Diseases (ICD-10).

Patients were included if they met the following inclusion criteria: 1) registered in the NPR with ICD-10 code C34.0-9 as the primary diagnosis, 2) diagnosed in the study period from 1 May 2010 to 31 August 2010, 3) living in Denmark, 4) ≥ 18 years and 5) listed with a Danish GP. We excluded patients who had previously been registered with any cancer type, except for non-melanoma skin cancer (C44), in the Danish Cancer Registry (DCR) [15]. The sampling of patients has been described in detail elsewhere [16].

### Data collection

A questionnaire was sent to the general practice at which the patient was listed. The questionnaire solicited information about the extent of the GP’s involvement in the lung cancer diagnosis and the key dates in the diagnostic process. Participating GPs were also asked to list the symptoms and signs presented by the patients and to describe how they interpreted these symptoms. The questionnaire was developed in 2009 by a group of researchers at the Research Unit for General Practice, Aarhus University [17]. As no pre-designed questionnaires for this specific purpose were available, ad hoc questions were constructed on the basis of previously used validated items [9,16,18]. In practices with several GPs, the GP who was most familiar with the patient was asked to complete the questionnaire on the basis of the medical records. Non-responders received a reminder after four weeks. No reimbursement was offered for participation.

### Data sources

We used the DCR to verify the diagnosis and to obtain data on tumour stage at diagnosis [15]. The DCR contains information about Danish cancer patients, including the date of diagnosis and tumour characteristics. DCR information on tumour characteristics at diagnosis rests on a multidisciplinary health-care team’s evaluation of either pathological (pTNM) or clinical (cTNM) data [19]. If any of the T, N or M values were missing, we categorised SCLC as ‘limited’ if the tumour was M0 (no metastases present) and as ‘extensive’ if the tumour was M1 (metastases present) regardless of the values (known or unknown) of other components. We categorised NSCLC as ‘advanced’ if the TNM stage included values of T4, N3 or M1 regardless of other components [20] (Table 1). To adjust for confounding by patient characteristics, we obtained data on comorbidity from the NPR; these data were based on ICD-10 codes for previous hospitalisations until the date of diagnosis. The presence of comorbidity was defined according to the Charlson Comorbidity Index (CCI) [21,22] and categorised as ‘low’ (CCI = 0), ‘medium’ (CCI = 1-2) or ‘high’ (CCI ≥ 3). Information about performed X-rays was also obtained from the NPR. Furthermore, we obtained socioeconomic information from the Danish Civil Registration System (CSR) [13] and the Danish Integrated Database for Labour Market Research (IDA).

**Table 1:** **TNM for NSCLC according to the 7th classification** [23]

**T/M**	**N0**	**N1**	**N2**	**N3**
**T1a** (≤2 cm)	IA	IIA	IIIA	IIIB
**T1b** (>2 cm)	IA	IIA	IIIA	IIIB
**T2a** (≤5 cm)	IB	IIA	IIIA	IIIB
**T2b** (>5 cm)	IIA	IIB	IIIA	IIIB
**T3** (>7 cm)	IIB	IIIA	IIIA	IIIB
**T3** (invasion)	IIB	IIIA	IIIA	IIIB
**T3** (same lobe nodules)	IIB	IIIA	IIIA	IIIB
**T4** (extension)	IIIA	IIIA	IIIB	IIIB
**T4** (pleural effusion)	IV	IV	IV	IV
**M1a** (ipsilateral lung)	IIIA	IIIA	IIIB	IIIB
**M1a** (contralateral lung)	IV	IV	IV	IV
**M1b** (distant)	IV	IV	IV	IV

The T component describes the extent of the primary tumour in terms of both size and local invasion. The N component describes regional lymph node involvement and the M component denotes whether distant metastases are present (M1) or not (M0).

### Variables

The patients were divided into groups depending on whether or not the GP responded to the questionnaire. GPs involved in the diagnosis were asked to state whether the patient had been referred through a fast-track route. Moreover, GPs were asked to rate their interpretation of presented symptoms as either 1) Alarm symptoms suggestive of cancer (alarm symptoms), 2) Symptoms suggestive of any serious illness (serious, but unspecific symptoms) or 3) Vague or ill-defined symptoms not directly suggestive of cancer or other serious illness (vague symptoms).

The primary care intervals and the diagnostic intervals were calculated by combining data from the DCR and the questionnaire. The primary care interval was defined as the time from the first presentation of lung cancer symptoms in primary care until referral to secondary care, and the diagnostic interval was defined as the time from first presentation until final diagnosis [24].

Cancer stage at diagnosis was classified according to the TNM system (version 6) and dichotomised into local and advanced disease. A cut-point between stage IIB and IIIA was chosen as previous studies have documented a significant difference in mortality between these two stages [25].

The socioeconomic variables considered in the study were education and marital status. Education included basic school and was dichotomised into ‘≤10 years’ and ‘>10 years’ [26]. Marital status was dichotomised into ‘cohabitating’ or ‘living alone’.

Comorbidity was assessed by using the Charlson Comorbidity Index (CCI); the index date was set as the day before the first contact with the GP (for patients for whom the GP was involved in the diagnosis) or the day before diagnosis (for patients for whom the GP was not involved in the diagnosis).

### Statistical analyses

Patient groups were compared using Wilcoxon’s rank-sum test for ordinal or continuous data, including time intervals, Kruskal-Wallis test for differences between groups or Pearson’s chi-squared test for nominal or dichotomous data. In the analyses for Table 2, patients with unknown characteristics were treated similarly to patients with known characteristics, but patients with unknown characteristics were excluded in the analyses for Table 3.

**Table 2:** **Characteristics of study population and patients with non**-**responding GP**

	**GP responders:**									
	**GP involved**		**GP not involved**		**P**-**value**	**All responders**		**Non**-**responders:**		**P**-**value**
	**N**	**(%)**	**N**	**(%)**		**N**	**(%)**	**N**	**(%)**	
**All**	**464**	**(68.3)**	**215**	**(31.7)**		**690**	**(71.1)**	**281**	**(28.9)**	
**Sex:**										
Male	265	(57.1)	110	(51.2)	0.147^1^	379	(54.9)	168	(59.8)	0.167^1^
Female	199	(42.9)	105	(48.8)		311	(45.1)	113	(40.2)	
**Age:**										
Mean	68.5		70.1		0.069^1^	69.0		69.7		0.174^1^
18-39 years	3	(0.7)	2	(0.9)	0.040^2^	5	(0.7)	2	(0.7)	0.461^2^
40-59 years	89	(19.2)	32	(14.9)		123	(17.8)	45	(16.0)	
60-79 years	306	(65.9)	136	(63.3)		448	(64.9)	184	(65.5)	
80+ years	66	(14.2)	45	(20.9)		114	(16.6)	50	(17.8)	
**Education:**										
≤ 10 years	207	(44.6)	111	(51.6)	0.082^2^	324	(47.0)	145	(51.6)	0.105^2^
11-15 years	186	(40.1)	78	(36.3)		266	(38.6)	106	(37.7)	
> 15 years	51	(11.0)	18	(8.4)		71	(10.3)	19	(6.8)	
Unknown	20	(4.3)	8	(3.7)		29	(4.1)	11	(3.9)	
**Marital status:**										
Cohabitating	276	(59.5)	110	(51.2)	0.042^2^	391	(56.7)	166	(59.1)	0.507^2^
Living alone	188	(40.5)	105	(48.8)		298	(43.2)	115	(40.9)	
Unknown						1	(0.1)			
**Comorbidity** ^**3**^ **:**										
0	283	(61.0)	82	(38.1)	0.001^2^	367	(53.2)	154	(54.8)	0.514^2^
1-2	143	(30.8)	91	(42.3)		240	(34.8)	99	(35.2)	
3+	38	(8.2)	42	(19.6)		83	(12.0)	28	(10.0)	
**TNM stage:**										
Localised	69	(14.9)	42	(19.5)	0.123^2^	114	(16.5)	62	(22.1)	0.042^2^
Advanced	395	(85.1)	173	(80.5)		576	(83.5)	219	(77.9)	

^1^Differences between groups were tested by Wilcoxon’s rank-sum test. ^2^Differences between groups were tested by the Kruskal-Wallis test. ^3^Charlson Comorbidity Index (CCI) from NPR (index date: day before diagnosis).

**Table 3:** **Primary care and diagnostic intervals** (**median in days**) **for lung cancer patients referred through the GP**

	**Primary care interval**						**Diagnostic interval**					
	**N**	**Median**	**IQI**	**P** **-value**	**PR** **(95% ** **CI) ** **Unadjusted**	**PR** **(95** **% CI) ** **Adjusted**	**N**	**Median**	**IQI**	**P** **-value**	**PR** **(95% ** **CI) ** **Unadjusted**	**PR** **(95% ** **CI) ** **Adjusted**
**All**	**429**	**7**	**0-** **30**				**442**	**29**	**12-** **69**			
Gender												
Men	239	7	0-29	0.171	1	1	248	29	10-69	0.546	1	1
Women	190	11.5	0-30		1.0 (0.7-1.5)	1.1 (0.8-1.6)	194	28	13-73		1.1 (0.8-1.6)	1.2 (0.8-1.7)
Age												
18-68 years	226	7	0-24	0.146	1	1	233	23	10-56	0.005	1	1
69+ years	203	12	0-37		**1.4** **(1.0-** **2.0)**	**1.5** **(1.0-** **2.1)**	209	34	14-87		**1.6** **(1.2** **-2.2)**	**1.4** **(1.0-** **2.1)**
Education^1^												
≤ 10 years	189	13	1-39	0.015	1	1	196	32	15-79	0.022	1	1
> 10 years	221	5	0-25		**0.7** ** (0.5-** **1.0)**	0.8 (0.6-1.1)	227	24	8-65		0.8 (0.6-1.2)	0.9 (0.6-1.2)
Marital status												
Living together	255	7	0-30	0.972	1	1	263	28	11-69	0.542	1	1
Living alone	174	8	0-29		1.0 (0.7-1.4)	1.0 (0.7-1.4)	179	30	13-69		1.0 (0.7-1.5)	1.1 (0.7-1.5)
Charlson’s Index^2^												
0	292	8.5	0-31	0.234	1	1	301	27	10-65	0.071	1	1
1-2	112	4	0-26		0.8 (0.5-1.3)	0.8 (0.5-1.2)	114	30	13-73		1.0 (0.7-1.6)	1.0 (0.6-1.5)
3+	25	17	0-50		1.5 (0.8-2.6)	1.3 (0.8-2.3)	27	61	17-106		**2.0 (** **1.3-** **3.4)**	**1.9** **(1.2-** **3.0)**
GP’s symptom interpretation												
Alarm	135	1	0-7	0.001	1	1	136	15	7-38	0.001	1	1
Serious	143	6	0-21		1.7 (0.9-3.29	1.6 (0.9-3.1)	148	29	9-67		**1.7** **(1.0-** **2.9)**	**1.6** **(1.0-** **2.7)**
Vague	149	28	10-62		**4.7** **(2.7-** **8.1)**	**4.8** **(2.8-** **8.2)**	156	47	21-89		**2.4** **(1.5-** **3.9)**	**2.4** **(1.5** **-3.9)**
Use of fast-track route												
Yes	178	7	1-23	0.783	1	1	179	23	11-52	0.019	1	1
No	245	9	0-37		**1.5** **(1.1** **-2.2)**	**1.7** **(1.1-** **2.4)**	256	34	12-88		**1.8** **(1.2-** **2.7)**	**1.8** **(1.2** **-2.8)**

Only GP-involved patients were included in the analyses. Adjusted and unadjusted associations for long intervals (4th quartile) are presented as prevalence ratios (PRs) with 95% confidence intervals (95% CI).

^1^Years of education. ^2^Index date: day before first contact to GP.

Effect of gender, age, education, comorbidity and marital status are mutually adjusted. GP’s symptom interpretation and use of fast-track are adjusted for gender, age, education, comorbidity and marital status.

Numbers in bold are significant results.

Backward cumulative curves for the dates of the latest and the second-latest X-ray before diagnosis and associated 95% confidence bands were drawn by applying a standard Kaplan-Maier procedure and normal approximation on a reversed time scale.

We used generalised linear models for the binomial family to calculate the associations between long intervals and gender, age, marital status, education, comorbidity, GP interpretation and use of fast-track referral. Long intervals were defined as the 4th quartile for the full study population. Prevalence ratios were preferred to odds ratios, which would tend to overestimate the associations as the prevalence of the outcome measure was above 20% [27]. Consequently, we chose the logarithm for the link function to facilitate direct estimation of prevalence ratios. Analysis of time intervals was restricted to patients in whose diagnosis the GP was involved. Estimates are presented with 95% confidence intervals (95% CIs) when relevant. Data were analysed using the statistical software Stata 12.0 (StataCorp LP, TX, USA).

### Ethics

The study was approved by the Danish Data Protection Agency (Ref. no.: 2010-41-4694) and the Danish Health and Medicines Authority (J. no.: 7-505-29-1484/1 and Ref. no.: 7-604-04-2/195/EHE). The study did not require approval from the Committee on Health Research Ethics of the Central Denmark Region nor did it require consent from individuals as no biomedical intervention was performed.

## Results

### Descriptive data

**Figure 1:**
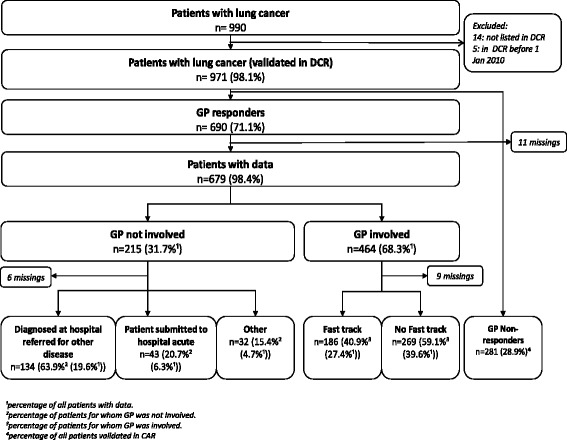
**Routes to diagnosis for consecutive primary lung cancer patients.**

A total of 990 lung cancer patients were identified in the NPR. We excluded 14 patients because the diagnosis could not be validated in the DCR. Five patients registered with a lung cancer diagnosis in the DCR before 1 January 2010 were also excluded. A questionnaire was sent to the GPs of the remaining 971 patients; 690 (71.1%) GPs responded (Figure 1). Patients listed with responding GPs had more advanced tumour stage at diagnosis (Table 2). Patients whose GPs were not involved in the diagnosis tended to be older and they were more likely to be living alone and to have a higher comorbidity score.

### Routes to diagnosis

GPs were involved in the diagnosis of 464 (68.3%) of the patients. Fast-track referral was used in 40.9% of the cases where a GP was involved. In total, 186 of the patients (27.4% of all patients in the study) started their route by presenting symptom(s) to the GP and subsequently being referred to the fast-track diagnostic pathway.

Patients whose GP was not involved in the diagnosis were most often diagnosed at the hospital to which they were referred for another disease (n = 134, i.e. 63.9%). In total, 43 (6.3%) of the patients were diagnosed in connection with an emergency admission (Figure 1).

### Diagnostic activity

**Figure 2:**
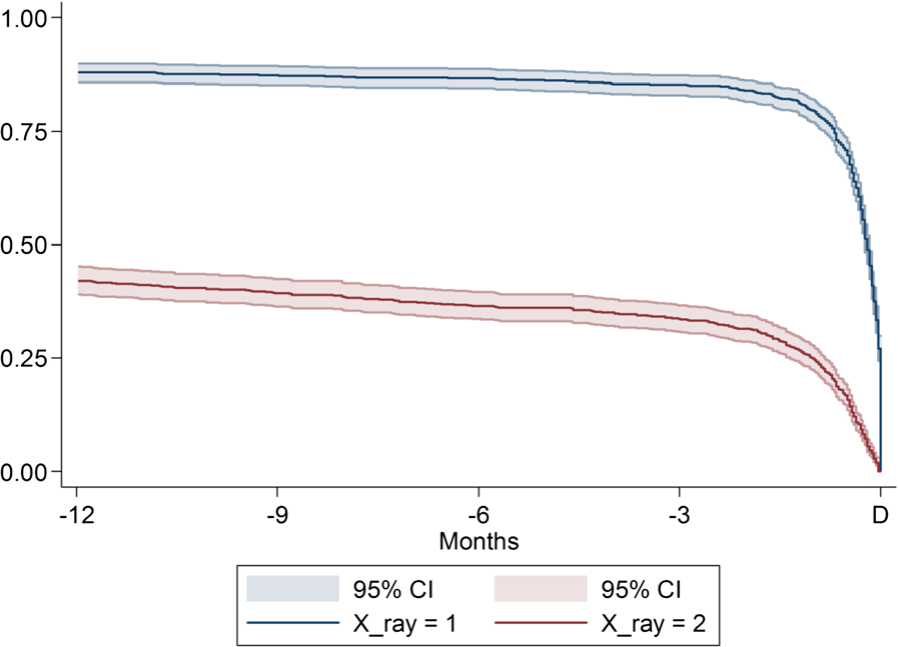
**Proportion of lung cancer patients with X**-**ray** **(s)** **performed during the 12 months immediately before diagnosis.** The upper curve (blue) shows the proportion of patients receiving at least one X-ray before diagnosis, while the lower (red) shows the proportion of patients receiving at least two X-rays before diagnosis. The curves should be read backwards from D (time for diagnosis), implying that approximately 34% of patients receive at least two X-rays 90 days before diagnosis. The bands are 95% confidence intervals.

Of all patients, 847 (87.2%) had at least one X-ray and 334 (34.4%) had at least two X-rays performed in the year preceding diagnosis. Most of the diagnostic activity occurred the last 90 days before the date of diagnosis (Figure 2).

#### Diagnostic activity 90 days prior to diagnosis

**Figure 3:**
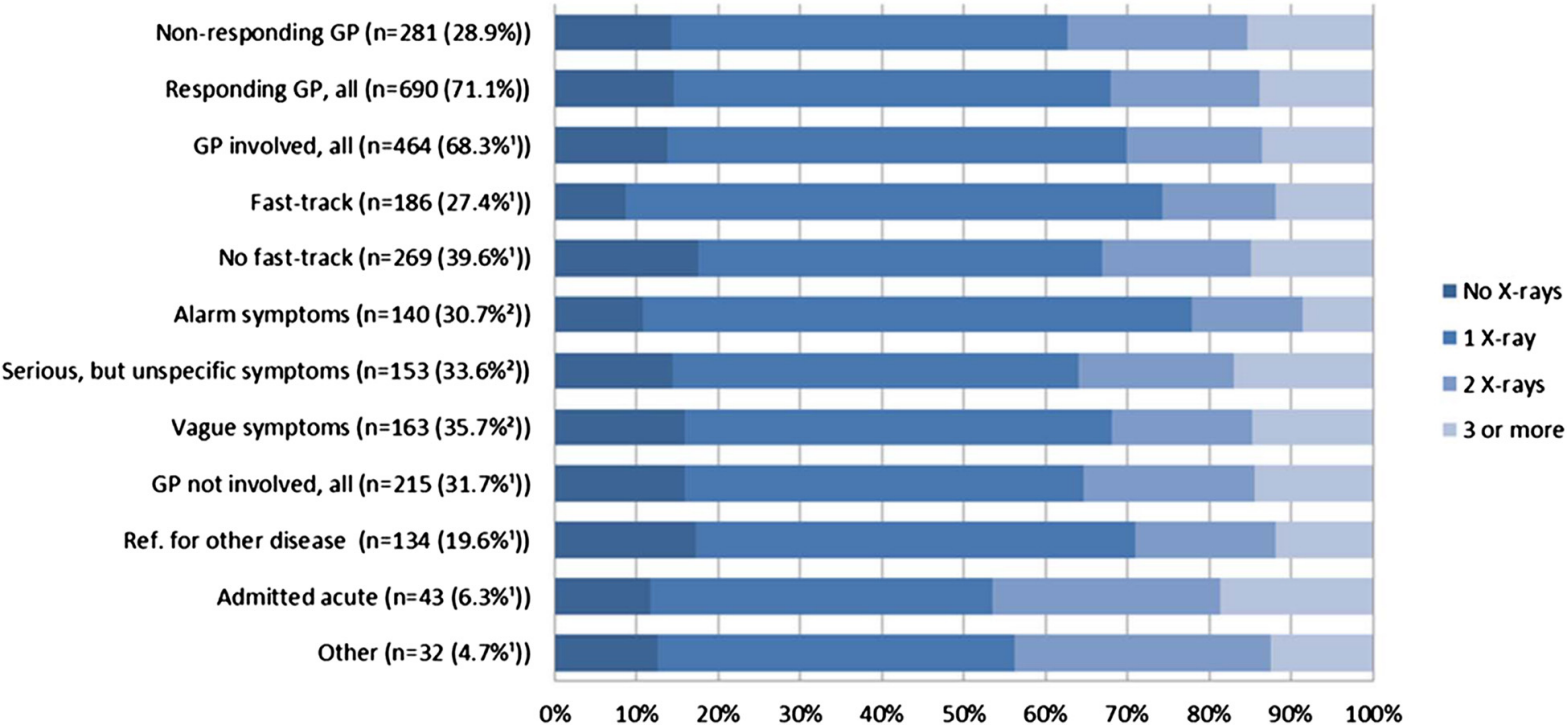
**Number of X**-**rays performed during the 90 days before diagnosis**, **depicted as percentages of patients with** **‘zero’,’** **one’,’** **two’** **or** ’**three or more’** **X**-**rays performed prior to diagnosis.** The columns refer to the patients in different routes to diagnosis as illustrated in Figure 1, except for the three columns with GP symptom interpretation. ^1^Percentage of all patients for whom the GP responded. ^2^Percentage of all patients for whom the GP was involved in the diagnosis (10 missings).

During the last 90 days before diagnosis, 831 (85.6%) of the patients had at least one X-ray performed and 326 (33.6%) had at least two. No differences were found in the number of performed X-rays between GP responders and GP non-responders (Figure 3) (p = 0.238) or between GPs involved in the diagnosis and non-involved GPs (p = 0.550). The proportion of patients who had one X-ray performed was higher among patients referred to fast-track (122 (66.0%, 95% CI: 58.3-73.4)) than among patients who were not referred to fast-track (133 (49.4%, 95% CI: 43.3-55.6)). The corresponding risk difference (RD) was 16.1 (95% CI: 7.1-25.2, p = 0.001) (Figure 3). The proportion of patients who had two or more x-rays was higher in patients whose symptoms were interpreted as ‘serious, but unspecific’ (35.9%, 95% CI: 28.4-44.1) than in patients whose symptoms were described as ‘alarm symptoms’ (22.1%, 95% CI: 15.6-30.0; RD 13.8, 95% CI: 3.6-24.1 (p = 0.010)). The proportion of patients who had two or more X-rays was higher among patients with co-morbidity (CCI > 0) (41.6%, 95% CI: 37.0-46.3) than among patients with no co-morbidity (CCI = 0) (26.8%, 95% CI: 23.2-30.8); RD 14.7, 95% CI: 8.8-20.6 (p = 0.001).

### Primary care interval and diagnostic interval

The overall median primary care interval was 7 days (interquartile interval (IQI): 0–30); the median diagnostic interval was 29 days (IQI: 12–69).

#### Patient-related factors

The median primary care interval and the diagnostic intervals were longer among patients with the lowest educational level (Table 3) than among better educated patients. Older age was statistically significantly associated with longer intervals of both primary care interval of >30 days and diagnostic interval of ≥69 days.

#### GP-related factors

The median primary care interval and the diagnostic interval were statistically significantly shorter if the GP suspected cancer or a serious disease (Table 3). Patients referred to a fast-track route experienced a statistically significantly shorter median diagnostic interval than patients not referred to a fast-track route. Patients with advanced disease stages had a statistically significantly shorter diagnostic interval than patients with localised disease, but, notably, not a longer primary care interval (median diagnostic interval: 26 vs. 40 days (p = 0.024), median primary care interval: 7 vs. 8 days (p = 0.462)).

A long primary care interval (adjusted PR: 4.8 (2.8-8.2)) and a long diagnostic interval (adjusted PR: 2.4 (1.5-3.9) were more likely if the GP interpreted the symptoms as ‘vague’ than if the GP interpreted the symptoms as ‘alarm’ symptoms (Table 3).

## Discussion

### Main findings

In a setting where GPs serve as gatekeepers to specialised medical care, including fast-track cancer diagnostic pathways, two thirds of lung cancer patients were seen in general practice before they were diagnosed with cancer; one fourth of identified lung cancer patients were diagnosed through the fast-track route.

The GP assessed the primary care interval to be longer than one month in one fourth of all lung cancer patients, and the diagnostic interval was above 69 days. The length of the diagnostic interval was associated with patient age, GP interpretation of symptoms and referral to the fast-track pathway.

The number of patients who had a chest X-ray was higher among those who were diagnosed through the fast-track pathway than among those who were not, which may indicate that GPs favour using the X-ray as an entrance to the fast-track pathway over basing their clinical assessment on symptoms alone. Patients who bypassed the fast-track pathway were more likely to have either none or more than two X-rays than referred patients. Furthermore, if the GP interpreted the symptoms as ‘serious, but unspecific’, the proportion of patients who had two or more X-rays was higher than if the symptoms were interpreted as ‘alarm symptoms’. This may imply that these patients are more difficult to diagnose and that the preceding diagnostic activity did not reveal the lung cancer. Moreover, almost half of the acutely admitted patients had two or more X-rays, which could indicate that these patients were seen and investigated in primary care, but that no cancer was identified.

Notably, we found that one third of the patients had at least two X-rays performed within the last 90 days before being diagnosed; this suggests that some of these patients could have had a false negative test. This finding definitely calls for further research to test whether the use of imaging with a higher sensibility for lung cancer implies that fewer tests are needed. We would recommend a randomised controlled trial on better access to a technologically upgraded investigation, e.g. low-dose CT.

### Strength and weaknesses

This study encompassed the entire population of consecutive, newly diagnosed lung cancer patients in Denmark identified through a valid hospital registry. The large number of included patients ensured a high statistical precision. The response rate of 71.1% is very satisfactory. If non-responding GPs were reluctant to respond because of long primary care intervals, our results are underestimating the actual intervals. GPs could also have chosen not to respond because they were not involved in the diagnostic pathway; the diagnostic intervals may then be shorter for these patients because they are diagnosed in hospitals in connection with investigation for another disease. This would imply that the overall intervals were overestimated.

Recall bias could be introduced when GPs knew that the patient was diagnosed with lung cancer because this knowledge could influence the GP’s assessment of e.g. the date of first presentation, the symptoms and their interpretation. To minimise this bias, the GPs were asked to use their electronic records and the discharge letters from the hospitals. If recall bias made the GP underestimate the length of intervals in relation to symptom presentation, such bias would tend to underestimate the association between the time intervals and non-alarm symptoms.

The date of diagnosis was defined as the day when the diagnosis was initially made in connection with hospitalisation or an outpatient visit. Thus, the diagnostic intervals are shorter than if we had used the date of histological verification of the diagnosis. However, as we wanted to examine the number of GP-initiated X-rays performed before the diagnosis, our definition of diagnostic interval increased the validity of the diagnostic procedure by being a truly pre-diagnostic activity.

Since we had no indication as to why X-rays were performed, we may have overestimated the diagnostic activity as some X-rays may have been performed for non-cancer reasons, e.g. due to pneumonia. Still, also in these instances, the GPs would intend to rule out the possibility of cancer.

Small cell lung cancer, which grows more rapidly than other lung cancers, comprised 8-10% of all cases. The diagnostic intervals for this type of cancer may be shorter than for other cancers as more alarm symptoms tend to be present than in non-small cell lung cancer. However, we did not stratify for this as the type is not known by the GP at presentation.

### Generalisability

We included a well-defined study population. However, non-response bias should be considered when generalising the results. Furthermore, the findings should be interpreted carefully in view of the differences in healthcare systems around the world, e.g. levels of gatekeeping, fast-track referrals and access to X-ray services.

### Comparison with other studies

Our finding is comparable to that of a British retrospective study of 220 lung cancer patients [28] of whom 61% were referred from primary care to specialist investigation. In line with our findings, another British study from 2012 found that 24% of lung cancer patients were diagnosed through fast-track referral [29]. However, inpatient evaluation was found to account for only 4%, whereas 39% of the patients were diagnosed through emergency routes. The differences could be explained by the algorithm used to identify pathways as we were able to detect if patients were already registered in a hospital-based pathway. This hypothesis is supported by a British study from 2007 reporting results similar to ours and including emergency referrals in 5% and fast-track referrals in 23% of the cases [12].

A British study [28] reported much longer primary care intervals than our study (52 (IQI: 7–243)), which can be explained by differences in study designs. We used a GP questionnaire, whereas the British study used research assistants to scrutinise the medical records for nine predefined lung symptoms. The impact of this difference in study design has also been shown for colorectal cancer [30].

A Danish study from 2006 [9], i.e. prior to the introduction of fast-track referral in Denmark, reported longer median intervals than our study, both in primary (29 (IQI:10–63)) and secondary care (58 (IQI:42–70)). This may indicate that the introduction of fast-track pathways and the increased focus on early cancer detection in Denmark has had some effect.

We found that 85% of all patients had an X-ray during the 12 months immediately before diagnosis. This figure is higher than the corresponding figure in a British study from 2005, where 164 of 247 (66%) lung cancer patients had at least one chest X-ray requested from primary care in the year before the diagnosis [11]. Of all lung cancer patients, 15% had no X-ray. The possible lack of diagnostic activity before the diagnosis of lung cancer could be attributable to patients not seeing the GP or patient and/or GP unawareness of symptoms. A British interview-based study found that patients extensively framed their symptoms of lung cancer as ‘normal features of lifestyle and ageing processes’ [31].

At least one third of patients had two or more X-rays during the three months preceding diagnosis, and some of these could have been negative X-rays. This finding confirms earlier research [9-11] and indicates a need for a more critical use of X-rays in patients suspected of having lung cancer. The number of patients who had an X-ray performed was higher among those who referred to a fast-track pathway than among other lung cancer patients, and this fact may cause concern. If the GPs primarily use the fast track after a suspicious X-ray, the rather large number of false negative X-rays could explain why so few patients are referred to the fast-track pathway, which may ultimately lead to delayed diagnosis.

## Conclusion

Lung cancer patients follow various routes to diagnosis, and only 25% of the included patients were diagnosed through the fast-track route from general practice. Although many patients are seen in general practice, existing cancer may not be identified quickly. Fast-track pathways do not seem sufficient to ensure and support earlier lung cancer diagnosis, wherefore additional pathways are needed. GPs must be provided with better tools for assessment of lung cancer risk and investigation of early symptoms. The large number of repeated X-rays in patients without alarm symptoms and with comorbidity makes a strong case for testing of better diagnostic tools (e.g. low-dose CT scans) in a primary care setting. Patients with lower socioeconomic status, elderly patients and patients with comorbidity tended to have longer intervals. Identification of these three target groups is important to ensure shorter clinical pathways in the future and for healthcare planning in general.
